# Supplementary material for: Genomic Evolution and Transcriptional Changes in the Evolution of Prostate Cancer into Neuroendocrine and Ductal Carcinoma Types
Source: Int J Mol Sci. 2023 Aug 12;24(16):12722. doi: 10.3390/ijms241612722 (PMC10454593; doi:10.3390/ijms241612722)
Supplement: Supplementary file 1 [file ijms-24-12722-s001.zip › ijms-2540874-supplementary.pdf]

1. Clustering of copy number corrected SNVs in case 1

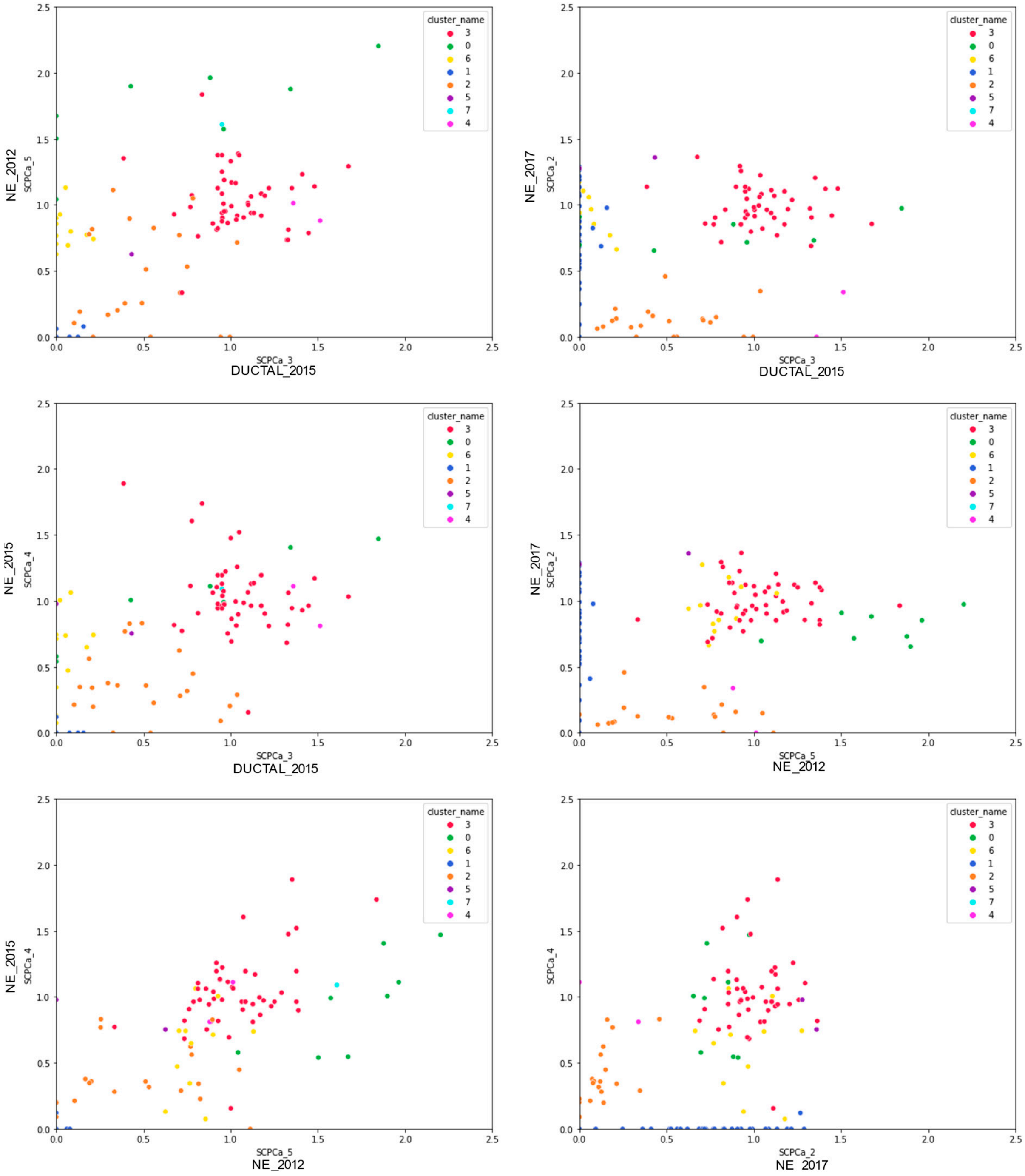

Figures S1: Pairwise plots of all samples from case 1 following Dirichlet Process clustering. Cluster 3 (corresponding to clone A in main Fig 3) was present in all samples at a cancer cell fraction of ~1 and hence was determined to be the most recent common ancestor. Cluster 1 (consisting of 34 SNVs, corresponding to clone B in main Fig 3) was present only in NE_2017. The remaining clusters were ignored as they were constituted by too few SNVs.

2. Differentially expressed genes in case 1

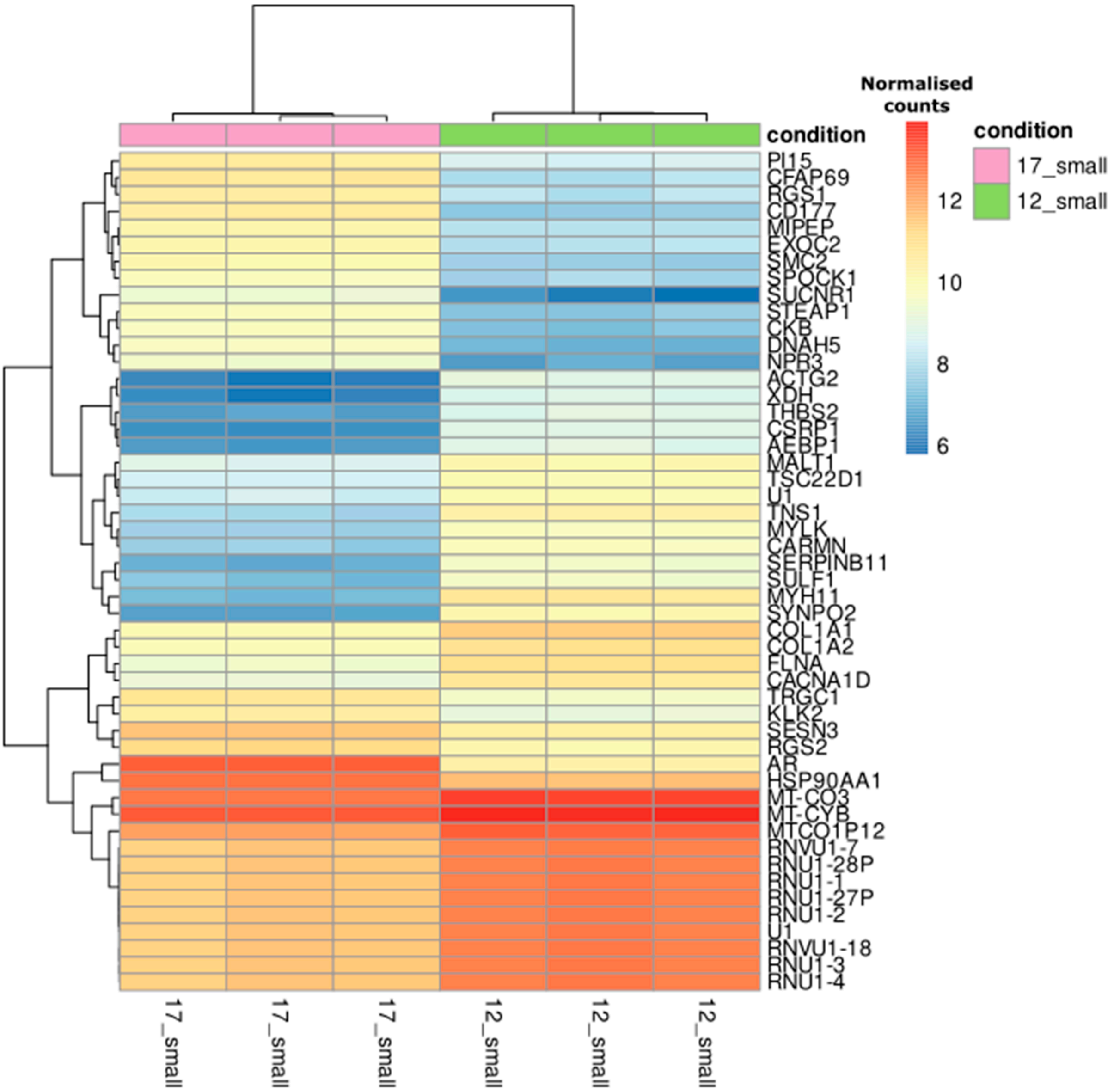

Figure S2: Heatmap of differentially expressed genes between NE_2017 and NE_2012 samples.

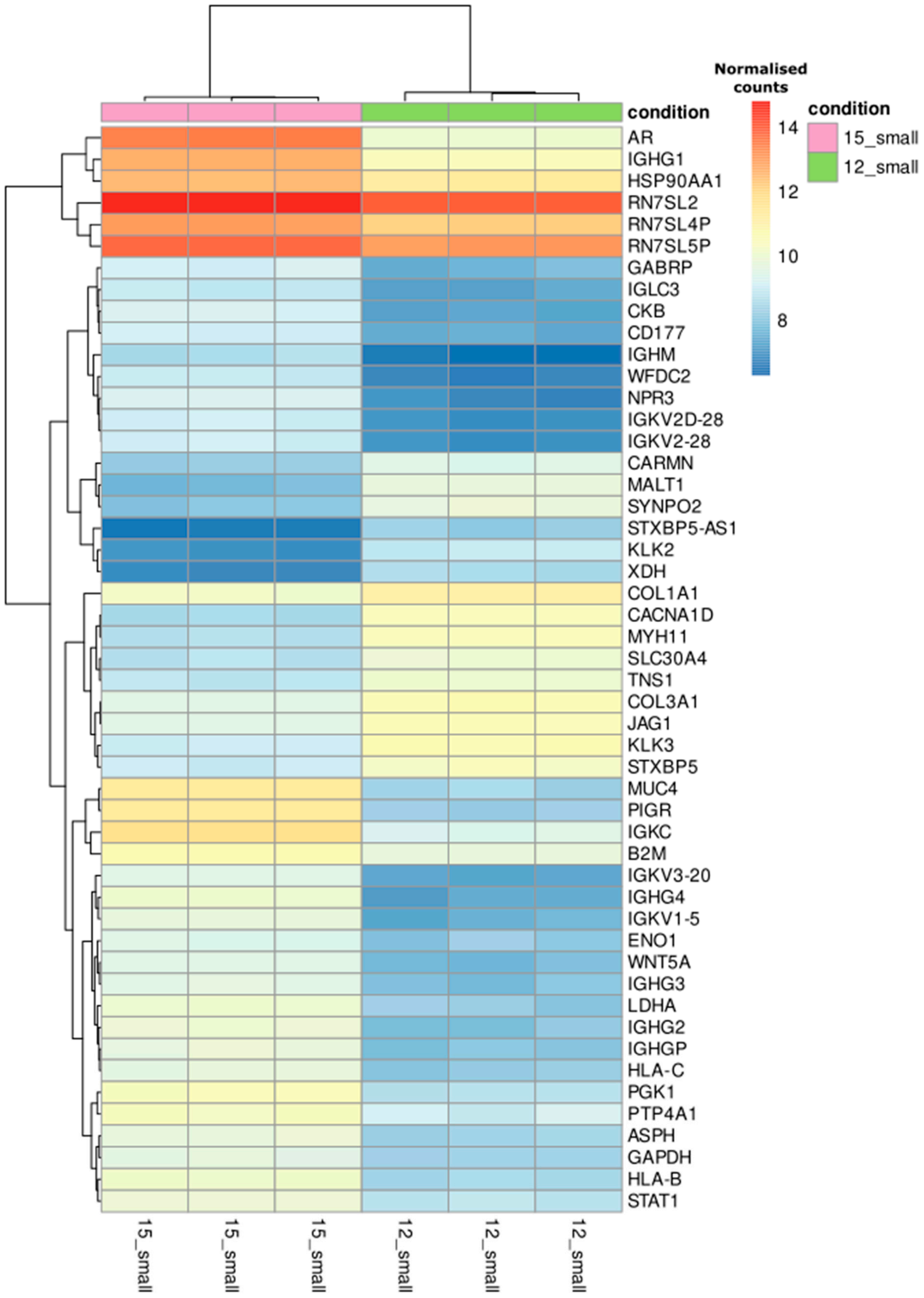

Figure S3: Heatmap of differentially expressed genes between NE_2015 and NE_2012 samples.

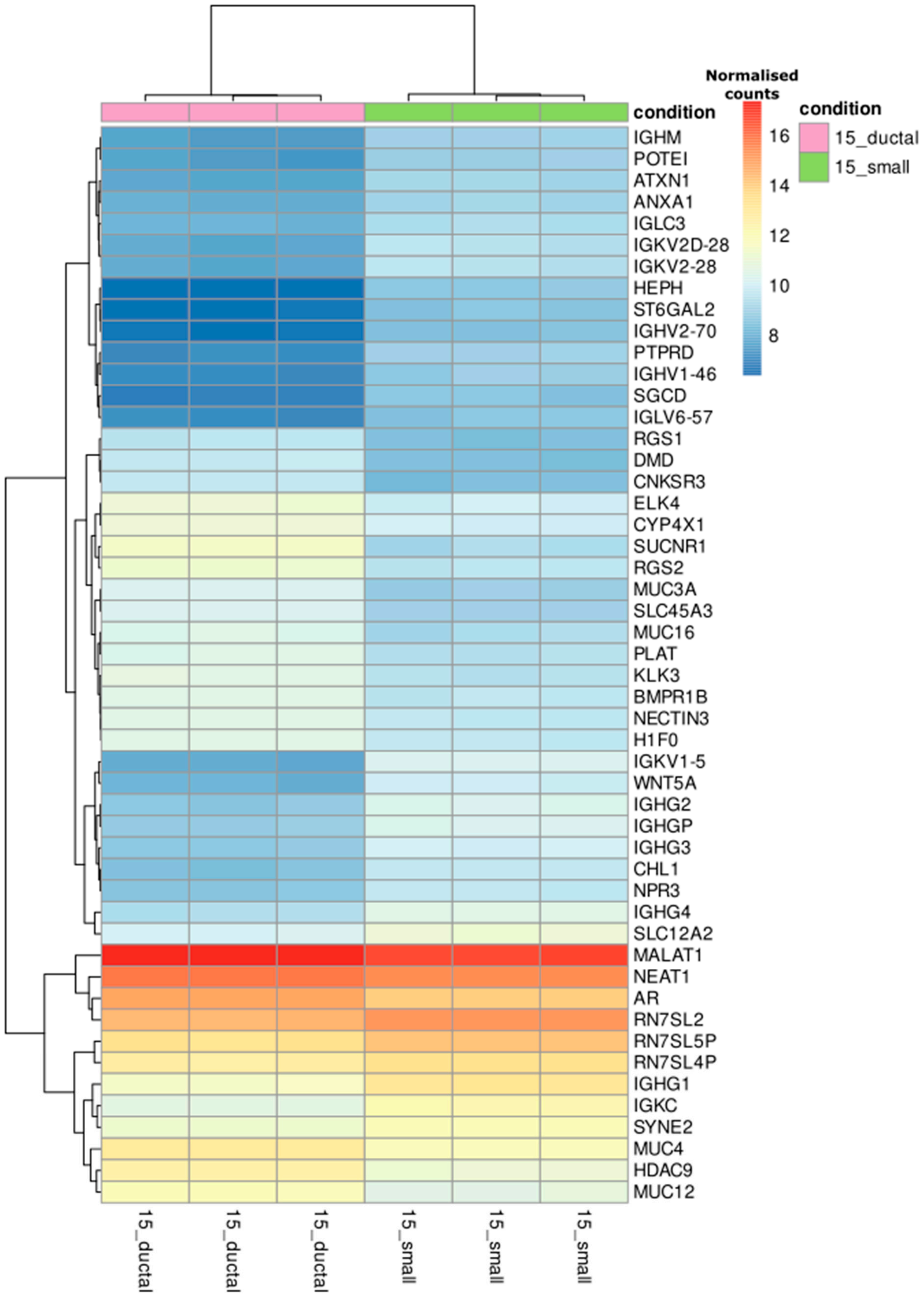

Figure S4: Heatmap of differentially expressed genes between DUCTAL_2015 and NE_2015 samples.

3. Clustering of copy number corrected SNVs in case 2

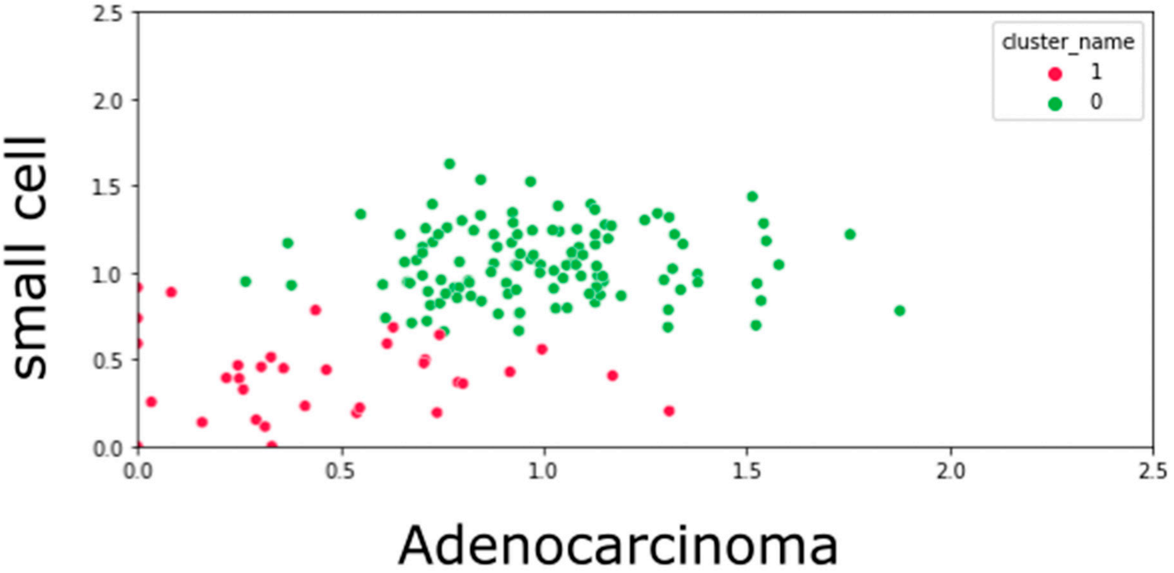

Figure S5: Pairwise plots of both samples from case 2 following Dirichlet Process clustering. Cluster 0 (corresponding to clone A in main Fig 5) is present at a cancer cell fraction of ~1 and hence was determined to be the most recent common ancestor.

4. Immunohistochemistry for the Androgen Receptor

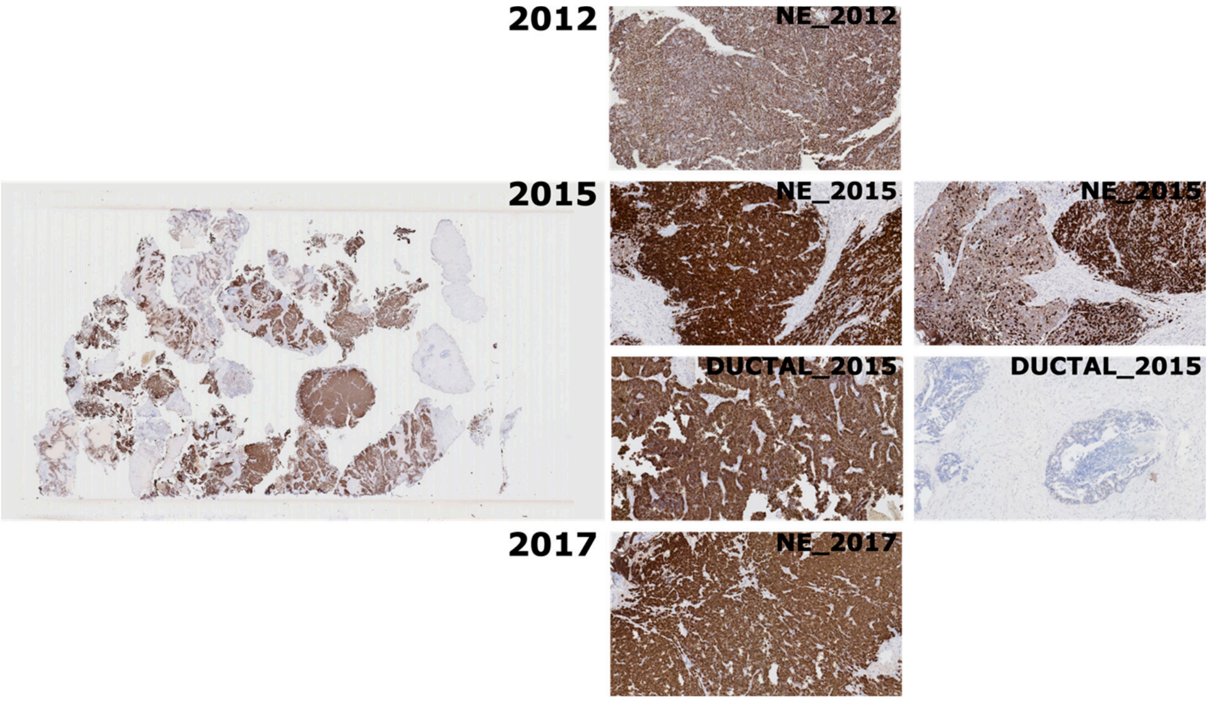

Figure S6: Immunohistochemistry staining for the Androgen Receptor for all samples from Patient 1. The image on the left is a low power view (0.5X) of the sample from the 2015 time point. The rest of the images are at 10X magnification, showing AR staining for the majority of cells in the regions (middle) and other areas with a different level of staining. While the AR expression is nuclear in NE_2012, in the rest of the samples (NE_2015, DUCTAL_2015, NE_2017) there is cytoplasmic staining in all cells where there is nuclear staining.
